# From “Me” to “We”: An Intervention Program to Increase Children’s Acceptance in Classrooms

**DOI:** 10.3390/children12121706

**Published:** 2025-12-17

**Authors:** Hannah Fisher Grafy, Yael Malin, Hagit Sabato

**Affiliations:** 1 Faculty of Education, Bar Ilan University, Ramat Gan 5290002, Israel; hamania10@gmail.com; 2 Max Planck Institute for Human Development, 14195 Berlin, Germany; 3 Seymour Fox School of Education, The Hebrew University, Jerusalem 9190501, Israel; hagit.sabato@mail.huji.ac.il

**Keywords:** peer acceptance, social status, school intervention, social interaction, latency phase

## Abstract

**Highlights:**

**What are the main findings?**

- Participation in the From “Me” to “We” intervention led to a small but statistically significant increase in children’s social status compared to a control group.
- The effect of the intervention was consistent across genders and independent of classroom-level differences.

**What are the implications of the main findings?**

- Group-oriented interventions targeting the shift from self-focused to collective per-spectives can support social acceptance in elementary classrooms.
- Future research is needed to explore long-term effects, implementation fidelity, and applicability across diverse classroom and cultural contexts.

**Abstract:**

Background: The elementary school years are a critical period for children’s social development, during which interactions with peers play a central role. While previous research highlights the importance of integration into the peer group, this study investigated the From “Me” to “We” school-based intervention, designed to enhance peer acceptance by encouraging children to move from an individualistic “Me” perspective toward a more group-oriented “We” mindset. Methods: Participants were 594 fourth- and fifth-graders (*Mage* = 9.57 years; 46% male, 54% female) from four schools in Israel. Students were assigned to an intervention group (*N* = 270) or a control group (*N* = 324). The From “Me” to “We” intervention, led by teachers with psychologist support, spanned three months and included six classroom sessions. Peer acceptance was evaluated using pre- and post-test social status questionnaires that assessed children’s actual social interactions with classmates. Results: Analyses revealed a significant interaction between the intervention and children’s acceptance within the group, such that among the intervention group, children’s social status significantly increased, whereas the control group showed no significant change. Conclusions: The From “Me” to “We” intervention led to a small but statistically significant increase in children’s peer acceptance. While these findings highlight the potential of group-oriented approaches to support social development in elementary school, future research is needed to examine the long-term effects and generalizability of the intervention across diverse classroom and cultural contexts.

## 1. Introduction

The elementary school years, also known as the *latency phase*, represent a crucial period in children’s social development. During this phase, children become more cognizant of their social environment [1,2] and acquire a more intricate understanding of their social context due to enhanced cognitive abilities and broader life experiences [3,4,5]. Early foundational theories in developmental psychology [6,7] have underscored the growing importance of peer relationships and social integration during middle childhood. They described it as a period in which children seek to develop a sense of competence through successful interaction with peers and highlighted the role of peer acceptance in shaping a child’s self-concept.

Social integration and assimilation necessitate the development of essential social competencies, including cooperative and synchronized interactions with peers, adherence to social norms, loyalty to the group, and active participation in shaping a collective group identity [8,9]. Accordingly, developmental research highlights a significant age-related increase in children’s internalization of societal norms and expectations [10,11], stronger identification with peer groups [12], and greater conformity with group rules and behavioral standards [9].

Recent studies have emphasized the critical role of egocentrism in shaping children’s social behavior. Higher levels of egocentrism have been linked to various maladaptive social outcomes, including increased online risk-taking among adolescents [13], reduced moral reasoning and theory of mind capacities in children [14], and lower social standing due to egocentric personality traits, particularly among early adolescents [15]. These findings suggest the need for a developmental perspective that unifies these elements into an understanding of children’s integration within their peer groups: the formation of a social self in parallel with the development of the group. This process requires a reduction in egocentrism and a gradual merging with the group, including its identity, social norms, shared loyalty, and collective cohesion.

The school environment is the primary social context where children undergo these developmental processes. The educational system requires significant effort and resources to create a positive school climate and ensure the students’ safety and welfare [16,17,18]. Within this context, educational teams face the mission of addressing children’s possible social rejection by peer groups and recognizing potential harmful consequences (present and future) for less accepted children and their peers [19,20,21,22].

Numerous school-based intervention programs aimed to reduce the probability of social rejection by promoting student well-being and fostering a positive classroom environment [23,24,25,26,27]. These programs typically rely on two complementary yet distinct theoretical approaches. The first is the social skills deficit model, which suggests that rejection arises from deficits in core social competencies, such as emotional regulation, conflict resolution, and peer-group entry [28]. School interventions aligned with this model focus on characteristics of less socially accepted children and identifying social–emotional deficits (e.g., rejection sensitivity, ADHD, externalizing behaviors, and impaired social skills) as primary factors leading to peer exclusion [29,30]. The second approach emphasizes the classroom’s social dynamics, wherein aggressive behaviors and peer rejection often result from high tension, conflict, and hierarchical structures within the group and lead to the marginalization of certain individuals as “scapegoats” [1,31].

This study evaluates the innovative *From “Me” to “We”* intervention, which aims to integrate both approaches by focusing on the child’s interactions within the group, alongside the group dynamics, while keeping in mind the social developmental task of integrating into the group. The intervention is grounded in the Social Reasoning Developmental (SRD) model [1,9], which focuses on the cognitive and moral dimensions of social behavior. This model suggests that children evaluate inclusion and exclusion decisions based on group loyalty, fairness, and identity [32]. According to the SRD perspective, the latency stage—a developmental period in which moral reasoning and group-related cognition emerge—is a crucial window for interventions. During this time, children begin to form their “social self,” which refers to the part of the self that reflects one’s participation in social groups [33] and are especially receptive to learning social skills and developing the capacity for group-based moral reasoning. The social self emerges through engagement with peers and is shaped by the dynamics of group membership. It may also involve a distinction between one’s public persona and private self [34]. The classroom peer group thus becomes the primary developmental context for shaping the social self during the latency period.

Accordingly, the *From “Me” to “We”* intervention targets children’s social status within their class as a main peer group and addresses social acceptance as a potentially inherent aspect of development during latency. The intervention aims to foster the development of the social self and to strengthen the interpersonal skills that support a positive classroom climate. From this perspective, peer acceptance is not only a desirable social outcome but also a developmental signal that children are engaging successfully in the key latency task: becoming integrated into the peer group [35,36,37].

This development task involves multiple domains, including cognition, perception, emotion, behavior, and morality, and requires children to shift from egocentrism to group-oriented functioning. The transition includes relinquishing control and omnipotence, reducing dependence on adults, and adopting group norms [38,39,40,41]. Ultimately, children are expected to attain “group independence,” a state in which they contribute meaningfully to the group while operating in sync with its norms and goals [36,37].

Children who are less socially accepted may struggle with this developmental progression. They often remain in a “Me” position, echoing the egocentric thinking described in Piaget’s early developmental stages [38,42]. These children may fail to align with class norms, acknowledge social hierarchies, reduce their reliance on adult validation, or integrate into the collective goals of the group [41]. As such, they may miss crucial opportunities for developing group-based moral reasoning and social competence.

The *From “Me” to “We”* intervention addresses group and individual dimensions. At the group level, it aims to foster a positive school climate, helping children and educators shape an optimal social self-object within the class. At the individual level, the program aims to facilitate a transformative shift in the internal perception of the children in the class. The intervention focuses on moving from a restrictive “Me” position, characterized by rigidity and self-centeredness, to a more inclusive “We” perspective. At the end of the program, children are expected to recognize themselves as a meaningful part of a group that assumes a central role in their world. This internal shift aims to empower children who are less socially accepted, enabling them to see themselves as valuable contributors to the group.

Previous research on change mechanisms in rejected children’s social status within the class (over a 2-year period) suggested self-perception as an important factor enabling children to improve their status among peers [43]. Thus, our intervention’s intended shift in children’s perspective from “Me” to “We” is crucial in initiating tangible change that helps children integrate into the group and engage and maintain social interaction with their peers. Thus, we expected the intervention to enhance their social status within the classroom over time. The program’s core modules offer every child an understanding of the choice between the “Me” position, which focuses on one’s self, goals, and interests, and the “We” position, which focuses on the group and its collective goals and shared interests. The modules expose the children to the advantages and disadvantages of each perspective.

Opting for the “Me” position allows the child to maintain a sense of individuality. However, at this developmental stage (when the group’s presence is crucial for personal growth), the “Me” position may also lead to social exclusion by group peers. Conversely, the “We” position entails sacrificing individual desires and ambitions but offers acceptance and integration within the group and elevated social status [36,41,44]. This emphasis on the individual’s perspective and transition to a position significantly identified with the group is expected to result in improved social status within the peer group from the individual’s perspective.

In the current study, we examine the *From “Me” to “We”* school intervention, which engages teachers and elementary school children in fostering a more inclusive classroom environment. Specifically, we assess the intervention’s impact on children’s social status by analyzing changes in their self-reported social interactions within their class before and after the program.

## 2. Materials and Methods

### 2.1. Participants

Our sample included fourth- and fifth-graders (*M_age_* = 9.57 years, *SD* = 0.55) attending four schools serving families of average socioeconomic backgrounds in the center of Israel. The four schools were randomly selected from a pool of 16 schools that expressed interest in the intervention, taking into account the resources available for implementing the program and conducting the research. We randomly assigned the classes within each school and grade level to the trial (intervention) and no-treatment control groups. Overall, 12 of 26 classes at the four schools took part in the intervention. We initially began with 756 participants. Of these, 48 students did not complete the pretest, 94 did not complete the post-test, and 20 did not complete both the pre- and post-tests. These participants were excluded from the analysis, resulting in a final sample of 594 students. All parents approved their children’s participation in the research. Participation in the research was not a requirement for joining the program. The sample characteristics by group present in Table 1.

The sample size was not determined a priori because it depended on the number of schools and classrooms willing to participate in the study. A sensitivity power analysis showed that with 594 participants, two groups, and 1 degree of freedom in the numerator, we had 85% power to detect a small to medium effect size of (*f* = 0.12).

### 2.2. Design

The intervention program consisted of six sessions with the class and two guiding meetings with the class educators. Data were gathered in two pretest sessions before the intervention (1 week apart in November 2022) and two post-test sessions after the intervention (1 week apart in April–May 2023).

### 2.3. Procedure

The intervention was proposed to schools as one of the Ministry of Education’s educational programs to improve class climate in elementary schools. Interested principals enrolled in their schools. In addition, we recruited 35 psychologists through the Educational Psychological Service and trained them to lead the interventions.

The Ministry of Education Ethics Committee approved the study (approval #10284) as part of a larger project. In the pre- and post-tests, we measured social status in class settings using a questionnaire where children self-reported their interactions with each child in the class.

### 2.4. Training Implementation

The intervention lasted 3 months and included individual and group meetings between the school psychologists and the homeroom teachers. The psychologists led the initial training and provided ongoing support. A teacher and a psychologist co-facilitated the classroom sessions as follows: Initially, all school teachers participated in a 2-hour introductory workshop, during which the psychologists presented the program’s theoretical foundations, including developmental aspects of the latency stage, social hierarchy, and social rejection, as reviewed earlier [2,3,4,5,6,7,41]. A second 2-h training session was held exclusively with the intervention class teachers. It focused on the program’s structure, guiding principles, and implementation strategies. The psychologist also held weekly one-on-one consultations with the teachers throughout the intervention and continued to support them for an additional 6 weeks to reinforce the integration of the program’s core principles.

The program modules in the first three sessions introduced the children in the classroom to the two social stances—“Me” and “We.” They learned to recognize when they or their peers acted from a “Me” perspective, characterized by self-focus, rigid insistence on personal desires, and limited consideration of others’ perspectives and feelings, versus the “We” perspective, which emphasizes shared group identity, mutual understanding, cooperation, and collective responsibility.

In the subsequent three sessions, the children engaged in structured group play that enabled them to actively explore and practice transitioning between the “Me” and “We” positions. These experiential activities supported the development of social awareness, allowing students to recognize the functional value of both self-oriented (“Me”) and group-oriented (“We”) perspectives. By navigating between individual expression and collective engagement, the children became more attuned to the emotional and relational consequences of their choices, fostering greater flexibility and intentionality in their social interactions. Table 2 provides an overview of the intervention sessions.

### 2.5. Social Status Measure

The Social Status Measure [45,46] is a self-report questionnaire that focuses on children’s actual interactions with peers. Specifically, the children are asked to indicate, for each child in their class, whether they typically play with that child during school breaks, meet that child after school, or tell that child personal things. A trained research assistant introduced herself to the class as a student who had come to learn how children think. She explained that the children’s participation was voluntary, and they could decide not to participate. She further assured the children that we would maintain everyone’s privacy and anonymity and that final lists would not include names or be revealed to anyone at their school.

To administer the Social Status Measure, the researcher wrote the names of all the children in the class and their assigned serial numbers for the experiment (per the class student list) on the board and provided the children with a short paper questionnaire. The children were asked to complete a table consisting of four columns: The first column listed the serial numbers of the children in the class, and the remaining three columns referred to the types of social interaction (i.e., playing during school breaks, meeting after school, and telling personal things). For each row—representing a specific classmate by serial number, to preserve the participants’ privacy—the children were asked to tick the appropriate response (Yes or No) in each column.

### 2.6. Analytic Strategy

A social status score was calculated based on the number of children the child reported playing with during breaks, meeting after school, or telling personal things (an average of the three). A linear mixed-effects model was conducted to examine the intervention’s effect on change in social status, using the difference between post- and pre-intervention scores as the dependent variable. The model included intervention group as a fixed effect and classroom as a random intercept to account for the nested structure of students within classrooms.

## 3. Results

### 3.1. Preliminary Results

The Social Status Measure scores were examined for normality using the Shapiro–Wilk test. As expected for this type of measure, which reflects naturally varying social connectivity among children, some deviations from normality were observed. Specifically, preintervention scores in the control group (*W* = 0.983, *p* < 0.001) and post-intervention scores in both groups (control: *W* = 0.981, *p* < 0.001; intervention: *W* = 0.977, *p* < 0.001) deviated significantly from normality. The preintervention Social Status Measure scores ranged from 0 to 31 (*M* = 8.27, *SD* = 3.70) and did not significantly differ between the intervention (*M* = 8.04, *SD* = 3.27) and control (*M* = 8.46, *SD* = 4.01) groups, *F*(1, 593) = 2.53, *p* = 0.112.

### 3.2. Main Results—Social Status Change

**Figure 1:**
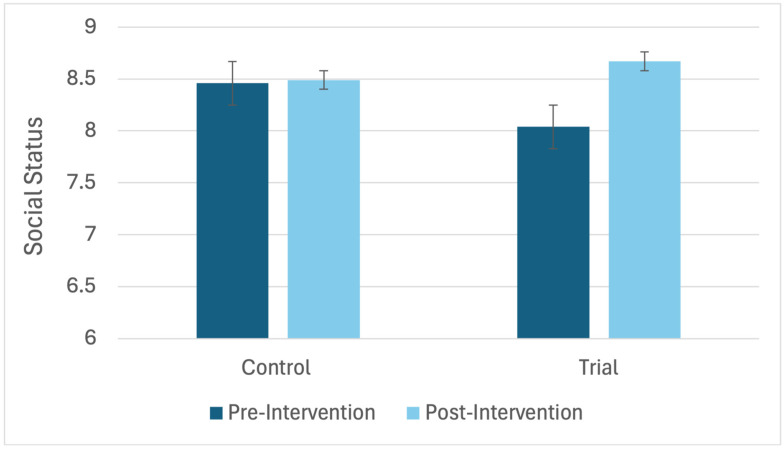
The Interaction Effect of the Intervention and Social Status (*N* = 594)**.** Bars represent the mean social interaction scores at pre- and post-test within each group. Error bars represent ±1 standard error of the mean.

A linear mixed-effects model with maximum likelihood estimation was conducted to examine the effect of the intervention on change in social status, while accounting for the nested structure of students within classrooms. The model included intervention group as a fixed effect and classroom as a random intercept. The results revealed a significant effect of the intervention on change in social status, *F*(1, 592) = 6.63, *p* = 0.010, indicating that children in the intervention group showed greater improvements than those in the control group. The intraclass correlation coefficient (ICC) was effectively zero, suggesting that virtually none of the variance in change scores was attributable to differences between classrooms. The standardized mean difference for change between intervention and control groups was *d* = 0.21, indicating a small effect. As illustrated in Figure 1, children in the intervention group increased in social status from pretest (*M* = 8.04, *SE* = 0.22) to post-test (*M* = 8.67, *SE* = 0.22; mean change = 0.63, 95% CI [0.35, 0.92]), whereas the control group remained largely stable (pretest: *M* = 8.46, *SE* = 0.20; post-test: *M* = 8.49, *SE* = 0.20; mean change = 0.03, 95% CI [−0.32, 0.38]). These findings indicate that the intervention produced a modest but statistically significant increase in children’s social status, independent of classroom-level clustering. 

### 3.3. Gender Differences

In an exploratory analysis, we examined gender differences in social status at baseline and in change scores following the intervention. At baseline, across both intervention and control groups, females (*M* = 8.48, *SD* = 3.74) and males (*M* = 8.01, *SD* = 3.64) did not differ significantly in social status, *F*(1, 592) = 0.24, *p* = 0.117. A mixed-effects model examining change in social status included intervention group, gender, and their interaction as fixed effects. The interaction between intervention group and gender was not statistically significant, *F*(1, 478) = 0.564, *p* = 0.453, indicating that the effect of the intervention on social status change did not differ between boys and girls.

## 4. Discussion

This study evaluated the *From “Me” to “We”* school-based intervention, which aims to enhance children’s acceptance within the classroom by encouraging a shift from self-focused to group-oriented perspectives. Using a randomized controlled trial, we assessed the program’s effectiveness among fourth- and fifth-grade students, a developmental period in which peer acceptance is particularly salient [19,20,22]. We examined whether participation in the program was associated with changes in children’s self-reported social interactions with classmates.

Participation in the intervention significantly improved students’ social status relative to the control group, providing preliminary evidence that the program may help promote greater social engagement. The effect size was small, reflecting a modest change rather than a substantial shift in overall classroom climate. Nevertheless, such effects are typical in classroom-based social interventions [47,48], where peer relationships are influenced by a wide range of individual, relational, and contextual factors. Importantly, the effect remained significant after accounting for classroom clustering, indicating that the observed improvement was not due to differences between classes.

Grounded in social developmental theories [1,9,35,36,37,40,41], the intervention conceptualizes the latency stage as a critical period for forming a group-based “social self.” Unlike traditional approaches that emphasize individual emotional or behavioral regulation [28,49], this perspective highlights the importance of social belonging, group flow, and synchronization with the group as developmental milestones. Within this framework, social status is viewed not as a static trait but as a reflection of a child’s ability to engage and align with group norms and values. Those who remain entrenched in a self-centered “Me” stance often experience rejection or marginalization, while those who adopt a “We” orientation gain status and inclusion [36,37,41].

Recent research underscores the relevance of egocentrism in understanding children’s social behavior. For instance, higher levels of egocentrism in adolescents have been associated with increased online risk-taking [13], lower levels of moral reasoning and theory of mind in children [14], and decreased popularity among peers due to egocentric personality traits [15]. These findings further support the *From “Me” to “We”* program’s focus on reducing egocentrism as a pathway to improved peer acceptance and classroom cohesion. By aligning developmental theory with classroom practice, this study presents a new framework for social–emotional learning that targets group belonging and collective identity as essential components of child development.

Previous interventions targeted the classroom social atmosphere (e.g., social rejection and bullying) by emphasizing personality characteristics [29,30,50]. Such interventions suggest improving children’s social–emotional skills (e.g., prosociality and empathy) by promoting a positive classroom climate [51]. Our intervention adds the novel component of the child’s perspective as the primary emphasis in changing the children’s social status. It focuses on the developmental task at the latency age—the transition from a “Me” to a “We” perspective. The program exposes children to the benefits and drawbacks of each perspective. They learn to mature and connect with the group while reducing egocentric tendencies. Whereas previous research stressed the child’s perspective as crucial in the process of social status change over time [43], our findings suggest a way to promote a change in the children’s perspectives, which affects their social experience among peers. It can potentially affect change at the class level and over the long term. However, future research should address these long-term effects.

Practically, our intervention targets an entire class, fostering group social skills among all students rather than focusing solely on less accepted children. For instance, in a conflict situation where a child in the “Me” position does not consider the “We,” the teacher presents both positions to the class, encouraging discussion and solution-finding among all group members. This attitude aims to promote a positive social climate in the classroom beyond its contribution to each child’s personal improvement within the group. Nevertheless, further research is needed to examine the long-term effects of this intervention on the class levels. Another benefit of our intervention is the teachers’ direct involvement in leading the program, enabling continuity and implementation of the core principles even after the program ends.

Despite our intervention’s positive outcomes, several limitations warrant consideration. First, social status was assessed using children’s self-reports of social interactions with class members. Although we asked children about their real behavior, reliance on self-reported sociometric questionnaires may introduce bias because the children’s self-perceptions might not accurately reflect their actual standing within the group. Future research could examine the intervention’s effects using other social status measurements (e.g., teachers’ reports). Second, the intervention was limited to six sessions due to practical constraints, which may have restricted the magnitude of observed effects. Future studies could explore longer or more intensive programs to determine whether increased exposure leads to larger or more durable gains. Third, the comparison group did not receive an attention-control condition, so some of the observed effects may reflect additional engagement rather than the specific content of the intervention. Future studies could include an attention-control group to isolate the specific impact of the program. Fourth, implementation may have varied across classrooms, with differences in facilitator style, teacher involvement, or classroom dynamics potentially affecting outcomes. Future research could monitor and standardize implementation more closely, or use fidelity measures to examine how variation in delivery influences effectiveness. Fifth, we did not empirically explore the long-term effects; a follow-up study is needed to examine the durability of change. Finally, the research scope should be expanded to include a diverse range of cultural and educational settings. This expansion could provide valuable insights into how the intervention can be adapted to different contexts, ensuring its broader applicability and effectiveness.

## 5. Conclusions

This study examined an intervention program aimed at improving children’s social acceptance within their classes by encouraging a transition from a “Me” to a “We” approach. The findings indicate that participation in the intervention led to a small but statistically significant increase in students’ social status, as reflected in participants’ self-reports on their interactions with peers. Given the study’s limitations, including reliance on self-reported measures and absence of an attention-control group, implications for the field should be considered preliminary and can be drawn more confidently only after future research addresses these limitations.

## Figures and Tables

**Table 1:** Participant Characteristics by Condition.

Group		Intervention *N* = 270	Control *N* = 324
Grade	Fourth	118 (43.7%)	137 (42.3%)
	Fifth	152 (56.3%)	187 (57.7%)
Gender	Females	120 (55.5%)	172 (53%)
	Males	150 (44.5%)	152 (47%)

**Table 2:** Overview of the intervention sessions.

Stage	Number of Sessions	Main Objective	Example Method
1	2	Introduce all schoolteachers to the program and core values	Teachers explore the developmental shift from an egocentric to a group-oriented perspective
2	2	Train intervention class teachers on program structure and co-facilitating sessions	Teachers participate in a simulation workshop, role-playing a full program session
3	3	During first three sessions with the class, introduce the children to the two social perspectives (“Me” and “We”)	Teacher reads story; students identify characters as displaying an egocentric (“Me”) or group-oriented (“We”) perspective and discuss the impact of each stance.
4	3	Allow children to practice applying these concepts through structured group play in the classroom’s social space and the schoolyard	Children play a group game, but those with egocentric mindsets disrupt the game. They are guided to understand their stance and to choose between a “Me” or “We” perspective

## Data Availability

The data for this study is available at the following OSF link- https://osf.io/m6zwr/?view_only=ad17efb5ffb74a39acae55a6dccd8282.
